# HLA-B27 Negative Reactive Monoarthritis of the Hip Joint Triggered by Scrub Typhus: A Case Report

**DOI:** 10.31729/jnma.8153

**Published:** 2023-05-31

**Authors:** Prabin Khatri, Ashutosh Upadhyaya, Nirajan Kandel, Shriya Upadhyaya, Himal Panth

**Affiliations:** 1 Department of Clinical Immunology and Rheumatology, Medanta Institute of Education & Research, Gurugram, Haryana, India; 2 Evergreen Hospital Pvt. Ltd., Parasi, Nawalparasi, Nepal; 3 Sangla Primary Health Center, Tarkeshwor, Kathmandu, Nepal; 4 Arogya Multispecialty Healthcare and Diagnostic Center, Bhairahawa, Rupandehi, Nepal

**Keywords:** *HLA-B27*, *case reports*, *reactive arthritis*, *scrub typhus*

## Abstract

Scrub typhus is common in rural Nepal's southern plains, but its diagnosis remains difficult due to a lack of clinical suspicion and poor diagnostic resources. The absence of common manifestations of the condition including eschar might further complicate this problem and may result in treatment delays. We report a case of scrub typhus with the primary presentation of reactive monoarthritis of the left hip joint in a 19-year-old male who presented with difficulty in walking, and pain over the left hip joint. Ultrasonography of the left hip and thigh showed features of synovitis and iliopsoas bursitis. After a meticulous workup, a diagnosis of human leukocyte antigen B27 negative reactive monoarthritis of the left hip joint triggered by scrub typhus infection was made and the patient was treated with doxycycline. Delays in treatment and the rate of complications can be prevented with high clinical suspicion and awareness of the atypical presentation of the condition.

## INTRODUCTION

*Orientia tsutsugamushiis* transmitted by the larva of the trombiculid mite. Perivascular inflammatory changes caused by the exaggerated Th1 response result in a variety of manifestations.^1,2^ Symptoms overlap with other tropical illnesses like malaria, dengue, brucellosis, and leptospirosis. Deranged hepatic enzymes, renal functions, and thrombocytopenia are seen in most patients with leukopenia or leukocytosis.^3^ Complications like acute respiratory distress syndrome (ARDS), meningoencephalitis, acute renal failure, and myocarditis are associated with increased mortality.^4^ Only a few cases of joint involvement in scrub typhus infection have been described in the literature. We present a rare case of HLA-B27 negative reactive monoarthritis of the hip joint triggered by scrub typhus, posing a diagnostic challenge.

## CASE REPORT

A 19-year-old male presented with difficulty walking, and pain in the left hip and around the left inguinal region. He had difficulty bearing weight on the left lower limb. He had a mild intermittent fever for 5-7 days, 2 weeks before the presentation. He had no rash, palpable purpura, pallor, icterus, or eschar. On examination, the left hip was held in an externally rotated position and there was restricted internal rotation, abduction, and flexion of the left hip. Enthesitis was noted at the insertion site of the iliopsoas on the lesser trochanter. No lesions were seen on the glans of the penis or the plantar surface of the heels. Eye and oral cavity exams were normal. There was no history of genitourinary or enteric symptoms and he has never been sexually active.

A complete blood count (CBC) revealed a leukocyte count of 14,000/mm^3^ with 80% neutrophils. Viral serology tests for human immunodeficiency virus (HIV), Hepatitis B Virus, and Hepatitis C virus were negative. Urinalysis, chest x-ray, renal function tests, malarial parasite antigen test, dengue antigen and antibody test, tuberculin skin test, anti-nuclear antibody, and rheumatoid factor were normal. C-reactive protein (CRP) was elevated. Ultrasonography (USG) of the left hip joint and thigh revealed fluid collection in the joint space with minimal thickening of capsules, a feature more consistent with synovitis than septic arthritis. Thickening of the iliopsoas bursa and well-defined thin-walled fluid collection along the line of the iliopsoas tendon was noted. There was no connection between the iliopsoas bursa and the hip joint, ruling out traumatic bursitis. The USG of the right hip joint was normal. Synovial fluid analysis revealed translucent fluid with a white blood cell (WBC) count of 23,000/mm^3^ (58% neutrophil). Gram staining revealed no organisms and the fluid was negative for crystals as well. Blood culture was also negative. An X-ray of the pelvis showed no evidence of sacroiliitis, but haziness was noted on the left lesser trochanter.

Serological tests for brucella, leptospira, scrub typhus, and HLA-B27 were ordered following no response to empiric antibiotic therapy. Immunoglobulin M (IgM) and immunoglobulin G (IgG) for scrub typhus were 1:240 and 1:320, respectively, while the rest of the tests, including HLA-B27, were negative. A diagnosis of HLA-B27 negative reactive monoarthritis of the left hip joint due to scrub typhus infection was made. The treatment regimen was then changed to Doxycycline and after receiving the drug for 5 days, the patient could ambulate. The patient had no complaints during his subsequent follow-up visits at 1, 3, and 6 months. IgM and IgG antibody titers for scrub typhus in the first follow-up were 1:32 and 1:48, respectively.

## DISCUSSION

Scrub typhus is a known cause of fever of unknown origin.^1^ But not much is known about the pathophysiology and virulence factors of this infection. It has been described to proliferate within small vessels of various organs after escaping the body's normal defenses. A milder form of the disease is associated with a balanced Th1/Th2 response, while severe forms show an exaggerated Th1 and suppressed Th2 response. The resulting vasculitis is considered to be responsible for the plethora of typical and atypical presentations of the disease.^2^ The incidence of eschar, maculopapular rashes, lymphadenopathy, gastrointestinal symptoms, hepatomegaly, and splenomegaly have been described in 7-68%, 30%, 23-93%, 40%, 66%, and 33% of cases, respectively.^1^ Eschar at the site of mite bite, though previously considered classical, may not always be observed. Increased vascular permeability and hypoperfusion of the affected tissue, lead to various complications like pneumonitis, encephalitis, acute kidney injury, myocarditis, and septic shock.

The Centre for disease control (CDC) recommends documenting a four-fold rise in antibody titer between acute and convalescent samples to diagnose scrub typhus.^5^ However, the consideration of scrub typhus as a differential diagnosis can be easily overlooked owing to its often-unusual presentations. In our case, septic arthritis was initially considered a differential but no significant findings on synovial fluid analysis led to vigorous work-up to find the cause. The absence of even remote history of urethritis and normal urinalysis made Chlamydia infection less likely. The presence of a high antibody titer for scrub typhus, joint effusions without hyper-echogenicity on ultrasonography, and extended discussion with orthopedics and radiology faculty favored the diagnosis of reactive monoarthritis due to scrub typhus infection. Prompt initiation of definitive treatment with close clinical monitoring resulted in favorable clinical outcomes in this case.

There is not enough literature describing reactive arthritis in the context of scrub typhus infection. Reactive arthritis is common in populations aged 20 to 40 years following urogenital infections like Chlamydia or Ureaplasma and enterogenic infections like Salmonella, Shigella, Yersinia, and Campylobacter. Key differential diagnoses include gonococcal arthritis, rheumatoid arthritis, septic arthritis, and other seronegative arthritis. In reactive arthritis, plain X-rays of the joints may show minimal nonspecific inflammatory joint findings like synovitis in the acute stage. Persistent synovial involvement is also one of the diagnostic criteria established by the American College of Rheumatology in 1999. Classic hallmarks such as floating osteophytes, sacroiliitis, erosions, enthesopathy, juxta-articular osteoporosis, homogenous joint space loss, and soft-tissue edema are seen in later stages.^6^ A paper published in 2020 explaining the role of USG in identifying arthritis in its early stages recommends that USG findings of synovitis and fluid collection are nonspecific lesions but the presence of collateral ligament inflammation and bursitis should raise suspicion for reactive arthritis. Meanwhile, hyperechoic or mixed aspects of the joint fluid are suggestive of septic origin.^7^

Reactive arthritis is sterile, inflammatory synovitis that presents within four weeks after being infected with organisms that have a predilection for mucosal surfaces. Asymmetric oligoarthritis involving large lower limb joints is frequently seen. About 80% of patients with reactive arthritis are HLA-B27 positive. Such patients frequently have systemic symptoms as well as extra-articular manifestations such as cutaneous, ocular, and enthesopathy.^8^ Although arthritogenic diseases are often limited to urogenital or enteric illnesses, the infection spectrum varies considerably. Stool and urine culture, serology for arthritogenic bacteria, or polymerase chain reaction (PCR) to detect bacterial DNA from synovium may be needed for diagnosis. Most cases of reactive arthritis are self-limiting without any significant sequelae, especially when it is HLA-B27 negative. HLA-B27 positivity is linked to an increase in disease severity, chronicity, and frequency of exacerbations, as well as the development of aortitis, uveitis, and spondylitis. A poor prognosis is attributed to factors such as reinfection, male gender, hip arthritis, erythrocyte sedimentation rate (ESR)>30 mm/h, sausage digits, poor response to non-steroidal anti-inflammatory drugs (NSAIDs), HLA-B27 positivity, and heel pain.^9^

Musculoskeletal and rheumatic features of infection by scrub typhus are usually limited to arthralgia and myalgia in most cases. Joint involvement is known to occur in many rickettsial diseases, but the same in association with scrub typhus has not been described very often in the literature. A case of acute severe monoarthritis was reported in a 4-year-old child in India in 2018.^1^ Similarly, a case of polyarthritis was reported following a scrub typhus infection in 2021.^10^ Adult-onset Still disease following scrub typhus infection was also reported in 2015 in Korea.^11^ Atypical presentations of the disease can often lead to delays in treatment, which may result in severe complications like ARDS, septic shock, and multisystem organ failure. The mortality rate was 9% in a recently published large cohort of 623 patients hospitalized with scrub typhus of varying severity, from mild to critically ill.^4^ Meanwhile, 20% mortality was found in another study among patients admitted to the intensive care unit (ICU).^12^

Scrub typhus with extra-articular symptoms is known to respond substantially to prompt treatment of Doxycycline. With the possible exception of Chlamydia-induced reactive arthritis, antibiotics are unlikely to affect the course of illness if a preceding infection has already triggered reactive arthritis.^8^ Meanwhile, in our case, Doxycycline treatment resulted in a speedy recovery of symptoms. Empiric use of this drug has been advocated in the literature, especially in endemic areas where the risk of mortality associated with lethal complications is high.^12^ The limited access to rapid tests and the low sensitivity of the tests, especially early in the disease course, highlights the importance of early clinical diagnosis of this curable illness. As it is transmitted by mites found in bushes and vegetation, mass education about preventive strategies like the application of insect repellents, which are treated with N, N-diethyl-meta-toluamide (DEET), and washing clothes in a 0.5% permethrin solution is also equally important.^3^

Reactive arthritis can be a presenting feature of scrub typhus and thus it must be considered as a differential, especially in endemic areas. As patients with scrub typhus have an excellent response to treatment, delays in treatment and complications can be prevented with high clinical suspicion and knowledge among clinicians about the atypical presentations of the condition.
